# Supplementary material for: Model discovery to link neural activity to behavioral tasks
Source: eLife. 2023 Jun 6;12:e83289. doi: 10.7554/eLife.83289 (PMC10310322; doi:10.7554/eLife.83289)
Supplement: Supplementary file 1. [file elife-83289-supp1.docx]

| **Abbreviation** | **Z-Brain Region Name** |
| --- | --- |
| AC | Telencephalon - Anterior Commissure |
| ARPS | Rhombencephalon - Area Postrema |
| AVTM | Rhombencephalon - Anterior Cluster of nV Trigeminal Motorneurons |
| CCRB | Rhombencephalon - Corpus Cerebelli |
| CHYP | Diencephalon - Caudal Hypothalamus |
| CRBL | Rhombencephalon - Cerebellum |
| DTHL | Diencephalon - Dorsal Thalamus |
| EMGR | Rhombencephalon - Eminentia Granularis |
| ETHL | Diencephalon - Eminentia Thalami |
| GAD1 | Rhombencephalon - Gad1b Stripe 1 |
| GAD2 | Rhombencephalon - Gad1b Stripe 2 |
| GAD3 | Rhombencephalon - Gad1b Stripe 3 |
| GLY1 | Rhombencephalon - Glyt2 Stripe 1 |
| GLY2 | Rhombencephalon - Glyt2 Stripe 2 |
| GLY3 | Rhombencephalon - Glyt2 Stripe 3 |
| HABN | Diencephalon - Habenula |
| IHYP | Diencephalon - Intermediate Hypothalamus |
| INFO | Rhombencephalon - Inferior Olive |
| IPN | Rhombencephalon - Interpeduncular Nucleus |
| ISL1 | Rhombencephalon - Isl1 Stripe 1 |
| LBCC | Rhombencephalon - Lobus caudalis cerebelli |
| LCOE | Rhombencephalon - Locus Coeruleus |
| LREN | Rhombencephalon - Lateral Reticular Nucleus |
| MTCB | Mesencephalon - Medial Tectal Band |
| MVNC | Rhombencephalon - Medial Vestibular Nucleus |
| NMLF | Mesencephalon - NucMLF (nucleus of the medial longitudinal fascicle) |
| NNIV | Rhombencephalon - Noradrendergic neurons of the Interfascicular and Vagal areas |
| OC | Telencephalon - Optic Commissure |
| OCN3 | Mesencephalon - Oculomotor Nucleus nIII |
| OCN4 | Rhombencephalon - Oculomotor Nucleus nIV |
| OLI2 | Rhombencephalon - Olig2 Stripe |
| PALL | Telencephalon - Pallium |
| PINE | Diencephalon - Pineal |
| PITU | Diencephalon - Pituitary |
| POA | Diencephalon - Preoptic Area |
| POC | Telencephalon - Postoptic Commissure |
| POCM | Diencephalon - Postoptic Commissure |
| PTCT | Diencephalon - Pretectum |
| PTM1 | Diencephalon - Migrated Area of the Pretectum (M1) |
| PTM2 | Diencephalon - Migrated Posterior Tubercular Area (M2) |
| PTUB | Diencephalon - Posterior Tuberculum |
| PVTM | Rhombencephalon - Posterior Cluster of nV Trigeminal Motorneurons |
| RCAD | Rhombencephalon - CaD |
| RCAV | Rhombencephalon - CaV |
| RH1 | Rhombencephalon - Rhombomere 1 |
| RH2 | Rhombencephalon - Rhombomere 2 |
| RH3 | Rhombencephalon - Rhombomere 3 |
| RH4 | Rhombencephalon - Rhombomere 4 |
| RH5 | Rhombencephalon - Rhombomere 5 |
| RH6 | Rhombencephalon - Rhombomere 6 |
| RH7 | Rhombencephalon - Rhombomere 7 |
| RHYP | Diencephalon - Rostral Hypothalamus |
| RPIN | Rhombencephalon - Raphe - Inferior |
| RPSP | Rhombencephalon - Raphe - Superior |
| SPAL | Telencephalon - Subpallium |
| SPOS | Telencephalon - Subpallial Otpb strip |
| TEGM | Mesencephalon - Tegmentum |
| TEM4 | Telencephalon - Telencephalic Migrated Area 4 (M4) |
| TOLO | Mesencephalon - Torus Longitudinalis |
| TOLT | Diencephalon - Torus Lateralis |
| TOSC | Mesencephalon - Torus Semicircularis |
| TSPV | Mesencephalon - Tectum Stratum Periventriculare |
| VCRB | Rhombencephalon - Valvula Cerebelli |
| VGL1 | Rhombencephalon - Vglut2 Stripe 1 |
| VGL2 | Rhombencephalon - Vglut2 Stripe 2 |
| VGL3 | Rhombencephalon - Vglut2 Stripe 3 |
| VGL4 | Rhombencephalon - Vglut2 Stripe 4 |
| VII | Rhombencephalon - VII Facial Motor and octavolateralis efferent neurons |
| VIIP | Rhombencephalon - VII' Facial Motor and octavolateralis efferent neurons |
| VMT1 | Rhombencephalon - Vmat2 Stripe1 |
| VMT2 | Rhombencephalon - Vmat2 Stripe2 |
| VMT3 | Rhombencephalon - Vmat2 Stripe3 |
| VTHL | Diencephalon - Ventral Thalamus |
| XVMN | Rhombencephalon - X Vagus motor neuron cluster |
